# Supplementary material for: Effect of Added Brewer’s Spent Grain on the Baking Value of Flour and the Quality of Wheat Bread
Source: Molecules. 2022 Mar 1;27(5):1624. doi: 10.3390/molecules27051624 (PMC8911926; doi:10.3390/molecules27051624)
Supplement: Supplementary file 1 [file molecules-27-01624-s001.zip › molecules-1615520-supplementary.pdf]

Spplementary Data

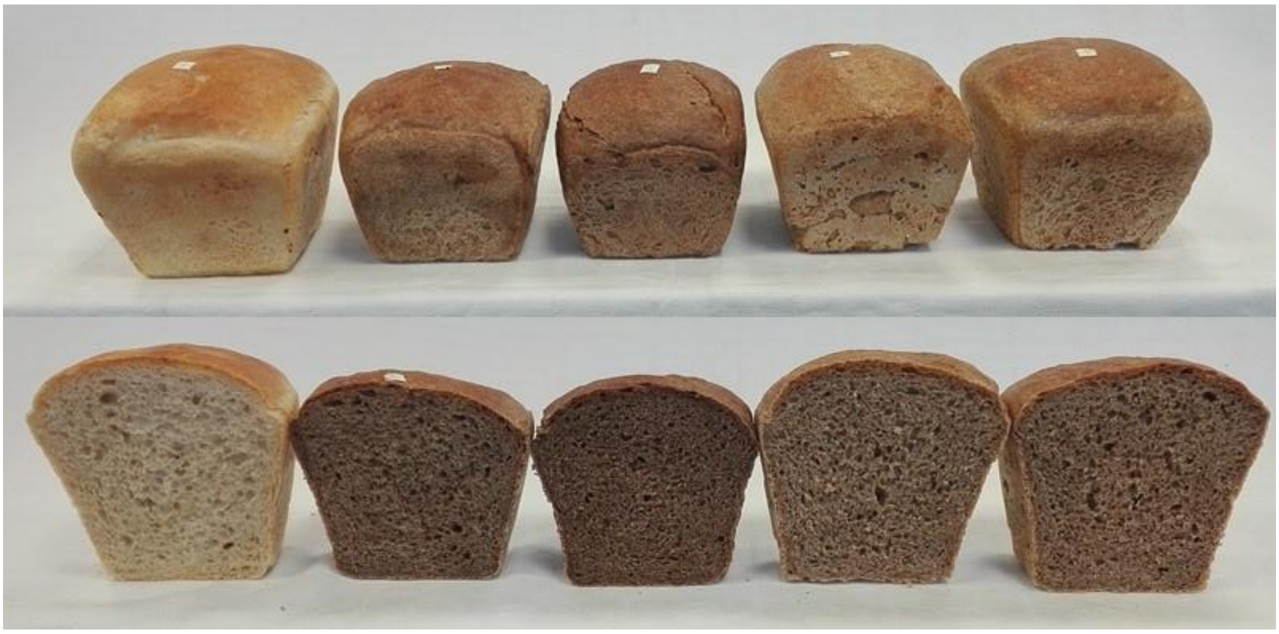

WF 10% 20% 10% 20%

BBSG BBSG+B

Figure S1. Wheat bread without and with BBSG and BBSG+B

Table S1. Significant Pearson's correlation coefficients (significance level $\alpha \leq 0,05$) between features of the protein complex and quality traits of bread

Traits	YB*	BV	SV	POC	SP	ZSI	WA	DDT	STAB
Yield of bread (YB)		-0,979	-0,990		-0,950	-0,935		0,907	
Bread volume per 100g flour (BV)	-0,979		0,997		0,901	0,903		-0,925	
Specific volume (SV)	-0,990	0,997			0,910	0,926		-0,936	
Porosity of crumb – Dallmann scale (POC)						-0,893	0,979		-0,939
Sum of points (SP)	-0,950	0,901	0,910						
Wet gluten (WG)									
Zeleny sedimentation index (ZSI)	-0,935	0,903	0,926	-0,893			-0,935		0,883
Water absorption of flour (WA)				0,979		-0,935			-0,923
Dough development time (DDT)	0,907	-0,925	-0,936						
Stability of dough (STAB)				-0,939		0,883	-0,923		
Degree of dough softening (SOFT)									

Table S2. Significant Pearson's correlation coefficients (significance level $\alpha \leq 0{,}05$) between amylose-starch complex and quality traits of bread

Traits		YB	BV	SV	POC	SP	FN	FT	MV
Yield of bread	(YB)		-0,979	-0,990		-0,950			
Bread volume per 100g flour	(BV)	-0,979		0,997		0,901	0,889		
Specific volume	(SV)	`	0,997			0,910			
Porosity of crumb – Dallmann scale	(POC)							0,933	-0,970
Sum of points	(SP)	-0,950	0,901	0,910					
Falling number	(FN)	-	0,889						
Initial gelatinization temperature	(IT)								
Final gelatinization temperature	(FT)				0,933				
Maximum viscosity	(MV)				-0,970				
